# Polypyrrole- and Polyaniline-Coated Cotton Fabrics as Efficient Adsorbents for the Pharmaceutical Water Contaminants Diclofenac and Salicylic Acid

**DOI:** 10.3390/polym15173563

**Published:** 2023-08-28

**Authors:** Hebatullah H. Farghal, Samar H. Tawakey, Wael A. Amer, Mohamad M. Ayad, Tarek M. Madkour, Mayyada M. H. El-Sayed

**Affiliations:** 1 Department of Chemistry, School of Sciences and Engineering, The American University in Cairo, AUC Avenue, New Cairo, P.O. Box 74, Cairo 11835, Egypt; hebatullahfarghal@aucegypt.edu (H.H.F.); tarekmadkour@aucegypt.edu (T.M.M.); 2 Chemistry Department, Faculty of Science, Tanta University, Tanta 31527, Egypt; samar.h.tawakey@gmail.com (S.H.T.); wael.amer@science.tanta.edu.eg (W.A.A.); or mohamad.ayad@ejust.edu.eg (M.M.A.); 3 Department of Chemistry, College of Science, University of Bahrain, Sakhir 32038, Bahrain; 4 Institute of Basic and Applied Sciences, Egypt-Japan University of Science and Technology, New Borg El-Arab City, Alexandria 21934, Egypt

**Keywords:** polypyrrole, polyaniline, cotton fabrics, diclofenac, salicylic acid, adsorption

## Abstract

The emerging pharmaceutical contaminants diclofenac (DCF) and salicylic acid (SA) pose potential hazards to humans and living organisms due to their persistence in water environments. In this work, the conductive polymers polypyrrole (PPY) and polyaniline (PANI) were successfully coated on cotton fabrics, as confirmed by FTIR and SEM measurements. The coated fabrics efficiently removed DCF at pH 5.3 and SA at pH 4, with removal efficiencies that exceeded 90% and 70%, respectively. Adsorption was rapid for most of the tested contaminant–fabric systems and reached equilibrium within 20–30 min. The best adsorption performance for both contaminants was shown on the PPY-coated fabrics, which yielded adsorption capacities of about 65 and 21 mg/g for DCF and SA, respectively. This could be explained by molecular modeling simulations, which mostly estimated higher total cohesive energy densities for adsorption on the PPY-coated fabrics than on the PANI-coated ones. The adsorption mechanism involved both coulombic electrostatic attractions and non-coulombic van der Waals and π-π stacking. The fabrics could be reused for three adsorption–desorption cycles. Immobilization of the conductive polymers on cotton fabrics provides a facile method for their handling and collection during adsorption and regeneration cycles while maintaining their multi-functionality in adsorbing different contaminants.

## 1. Introduction

Various pharmaceutical emerging contaminants have been recently detected in wastewater effluents of pharmaceutical industries, hospitals, and households due to the growing human consumption of therapeutics and personal hygiene products [1]. Diclofenac (DCF) and salicylic acid (SA) are two over-the-counter pharmaceuticals that are classified as non-steroidal anti-inflammatory drugs. They are used extensively and could pose health hazards. DCF is prescribed as an anti-inflammatory and analgesic for arthritis and acute injuries, and it can be administered topically or orally. It is a persistent drug in wastewater due to its poor degradation and high consumption rates, which led to ecological risks and tissue damage to mussels at concentrations of nanograms per liter [2]. The concentration of DCF in industrial wastewater in Pakistan amounted to 836 µg/L, as detected by HPLC-UV [3]. Regarding SA, it is a keratolytic agent that is used in ichthyosis and psoriasis and is difficult to remove from wastewater using conventional methods [4]. Moreover, it causes neurotoxicity and impaired respiration rates in *Mytilus galloprovincialis* [5]. SA was detected at a concentration of 27.8 mg/L in a North American wastewater treatment plant [6].

Several methods have been deployed for water treatment, such as coagulation and flocculation, membrane filtration, and advanced oxidation processes. However, coagulation and flocculation result in large amounts of sludge production, membrane technologies suffer from fouling and clogging of membrane pores, and advanced oxidation processes may result in the production of toxic byproducts [7]. On the other hand, adsorption is considered one of the most appropriate and facile methods for the removal of water pollutants in general and pharmaceutical drugs in particular since it operates efficiently without producing toxic substances [8,9,10]. A wide variety of adsorbent materials were developed for the removal of pharmaceutical drugs from wastewater [11]. Conducting polymers, including polyaniline (PANI) and polypyrrole (PPY), have attracted much attention in many applications due to their promising and unique properties, such as their large specific surface area, controllable pore size, and environmental and mechanical stability. Both PANI and PPY possess the basic requirements to act as efficient adsorbents owing to their stability in severe media, including high temperatures and aggressive chemical environments that allow thermal and chemical sterilization and recycling [12,13,14]. Nanotubular PANI was prepared with the soft-template method and applied for the dual removal of cationic and anionic dyes from wastewater, yielding good maximum adsorption capacities of about 57 and 69 mg/g for Acid Green and Methylene Blue dyes, respectively [13]. Macroporous PANI-poly(vinyl alcohol) aerogel was employed as an adsorbent and reductant for hexavalent chromium ions in aqueous solutions, and a remarkable adsorption capacity was obtained [15]. For environmental remediation and economic viability purposes, textiles and fabrics supported with different materials or polymers were applied in different fields. To attain easy separation and recyclability, PPY was deposited onto cotton fabric and decorated with silver nanoparticles for efficient and catalytic removal of *p*-nitrophenol from water [16]. Recently, PPY-coated cotton textiles were applied efficiently for the removal of methylene blue and Acid green 25 from aqueous solutions [17]. PPY and PANI were applied as coating materials for sawdust aiming to decolorize paper-mill wastewater, where removal efficiency reached 91.8 and 83.3%, respectively, using a 10 g/L adsorbent dose at 30 °C [18]. PPY was also grafted at different concentrations on coconut-shell-derived carbon to adsorb methyl tert-butyl ether and adsorption obeyed Langmuir isotherm, with maximum adsorption capacities (*q*_m_) ranging from 0.38 to 3.04 mg/g at 30 °C [19]. PANI-grafted chitosan was applied for the removal of lead (II) and cadmium (II) ions from aqueous solutions. According to the Dubinin–Radushkevich isotherm model, *q*_m_ reached 13.8 and 11.3 mg/g at pH 6, respectively, and the adsorbent was regenerable for three cycles using 0.1 M HNO_3_ [20].

Although many hybrid materials with quite interesting features were proposed for the removal of drugs from wastewater, most of them did not show impressive performance [21]. To the best of our knowledge, the application of PPY- or PANI-coated fabrics in the removal of pharmaceutical drugs from aqueous solutions has not been reported in previous literature. Pharmaceutical contaminants are known for their complex structures in addition to their bioactivity, which could negatively impact the health of living organisms when applied for prolonged times even at small doses [22]. Pharmaceutical compounds of the same therapeutic class do not always show the same behavior in the environment; they could have varying structures and thus different behavior towards applied adsorbents [23]. Herein, PPY- or PANI-coated cotton fabrics are applied for the first time to remove two drugs of the same therapeutic class, DCF and SA, from aqueous solutions. Deploying these proposed adsorbents in water treatment systems should aid in mitigating the harmful effects resulting from the accumulation and persistence of the two drugs in water streams. In addition, having the adsorbents in fabric form facilitates their handling and separation during operation. The coated fabrics are characterized by different methods such as Fourier transform infrared spectroscopy (FTIR), dynamic light scattering, and scanning electron microscopy (SEM) and are tested for the adsorption of DCF and SA in aqueous solutions. The adsorption kinetics of the drugs onto PPY- and PANI-coated cotton fabrics and the regeneration of the fabrics are also studied and discussed. Molecular modeling is applied to elucidate the interaction mechanism.

## 2. Experimental Procedure

### 2.1. Materials

Pyrrole (Sisco Research Laboratories, Mumbai, India, 99%) was purified via passing through an alumina column, and then the distilled pyrrole was kept at 5 °C for experimental use [24]. Iron(III) chloride hexahydrate (Alpha laboratories, Hampshire, UK, 98%) and ethanol (ADWIC, Cairo, Egypt, 99%) were used as received. Aniline (Acros Organics, Geel, Belgium, 99.8%) was purified by distillation over zinc powder and stored at 4 °C [25]. Distilled water was used for all preparation procedures. Diclofenac potassium salt was supplied from Sigma Aldrich (Sigma-Aldrich, Schnelldorf, Germany, purity: 99.8%). SA extra pure (99%) was bought from Oxford, India. Sodium hydroxide was bought from Fisher Scientific (Loughborough, UK), while HCl (37%) was bought from Acros Organics, Geel, Belgium.

### 2.2. Preparation of PPY- and PANI-Coated Cotton Fabrics

A sheet of Egyptian cotton textile (30 × 20 cm^2^) was soaked in a pyrrole monomer solution (0.2 M) for 4 h to allow complete impregnation of pyrrole on the textile. An equal volume of iron (III) chloride hexahydrate solution (0.5 M) was then added to the previous solution. The resulting solution was kept under gentle stirring for 2 h and then left at room temperature (25 ± 2 °C) for 24 h. Afterward, the PPY-coated cotton textile was removed and washed with a dilute HCl solution (0.1 M) and ethanol to remove any adhered PPY on the textile surface. The PPY-coated textile was finally air-dried at room temperature overnight.

**Figure 1:**
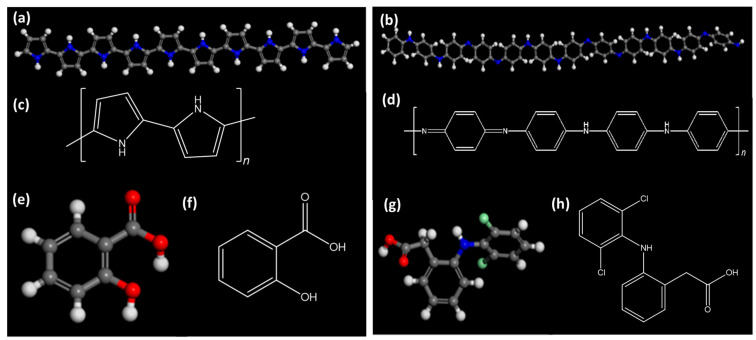
The 3D and 2D chemical structures of PPY (**a**,**c**), PANI (**b**,**d**), SA (**e**,**f**), and DCF (**g**,**h**).

For coating cotton with PANI, a sheet of Egyptian cotton textile was soaked in an aniline solution (0.2 M in 0.1 M HCl) for 4 h to allow the textile to become saturated with the aniline monomer solution. Afterward, 0.25 M of ammonium persulfate (APS) solution (prepared in 0.1 M HCl) was added to the saturated cotton solution, and the mixture was kept at gentle stirring for 2 h then was left for 24 h. The PANI-coated cloth was washed with a dilute HCl solution and ethanol and then left to air-dry at room temperature. The chemical 3D and 2D structures of PPY, PANI, SA, and DCF are shown in Figure 1.

### 2.3. Characterization

To determine the morphology of the coated fabrics, SEM imaging was performed on the Neoscope JCM-6000 Plus, JEOL Benchtop SEM, Tokyo, Japan. Also, FTIR measurements were performed on Thermo Scientific Nicolet 380, (Waltham, MA, USA), to determine the present functional groups in the range of 4000 to 650 cm^−1^ using the KBr disc method. Zeta potential measurements were performed on a Malvern Panalytical Zeta-sizer Nano Series Nano-ZS90 (Malvern, UK) to determine the fabric charge at pHs between about 3 and 11. The zeta potential was measured by cutting the fabric into small pieces (~3 × 3 mm), placing it in deionized water, and then adjusting the pH using 1 wt% NaOH or 0.5 M HCl. After stirring, fabric pieces were placed into the cuvette designated for zeta potential measurements [26,27].

### 2.4. Adsorption and Regeneration Studies

Batch adsorption studies were performed on a rotary shaker at 60 rpm to assess the effect of pH, contact time, adsorbent amount, and initial concentration on the adsorption capacity and removal efficiency of DCF and SA. Since the adsorbent is in the form of sheets, it was cut into uniform pieces, 1 × 2 cm^2^ each. The mass of each piece was in the range of 0.022 to 0.03 g and 0.015 to 0.0189 g for PPY- and PANI-coated fabrics, respectively. The volume of all solutions during adsorption was 15 mL. To study the effect of pH, DCF and SA adsorption was conducted at an initial concentration of 50 ppm and at a room temperature of 25 ± 2 °C using pHs of 5.3, 6.3, and 7.5 for DCF and 4.0, 6.3, and 7.5 for SA. For contact time profiles, three initial concentrations were applied at pH 5.3 for DCF adsorption on PPY- and PANI-coated fabrics and pH 4.0 for SA adsorption on each of the PPY- and PANI-coated fabrics. Adsorption was also studied at various surface areas (0.5–2.5 cm^2^) of the adsorbent at the optimal pHs mentioned previously and at an initial concentration of 50 ppm and 25 ppm for DCF and SA, respectively. The effect of initial concentration was performed with the previously mentioned parameters at a range of 10–50 ppm. After adsorption, the supernatant was obtained by decantation and measured on a UV/VIS spectrometer (Pg instruments, T80+ spectrometer, Leicestershire LE17 5BH, England) at 276 nm and 296 nm for DCF and SA, respectively. The measured absorbances were substituted in a calibration curve to obtain the equilibrium concentration of the adsorbate in milligrams per liter, and *C_e_* was used to calculate the percent removal and adsorption capacity using Equations (1) and (2), respectively.

(1) $$\%\;Removal=\frac{\left(C_{0}-C_{e}\right)}{C_{0}}\times 100$$

(2) $$q_{e}=\frac{\left(C_{0}-C_{e}\right)\;V}{m}$$

where *m* and *V* are the mass of the adsorbent in g and the volume of the adsorbate solution in L, respectively, whereas *C*_0_ is the initial concentration of the adsorbate in mg/L.

Regeneration studies were performed using 50 mL of 0.1 N HCl and 0.1 N NaOH as the desorbing agents for DCF and SA, respectively, after performing the adsorption experiment at 25 ppm of DCF or SA for 30 min at pHs of 5.3 and 4, respectively. However, due to the slow rate of SA adsorption on PPY-coated cloth, it was carried out for 4 h. Regeneration was carried out for three consecutive cycles.

### 2.5. Modeling Investigation

**Figure 2:**
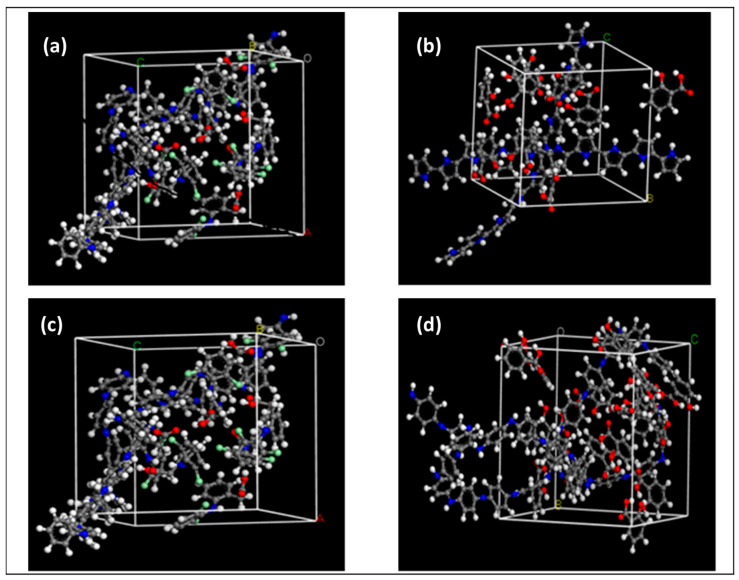
Simulation boxes for DCF on PPY (**a**), SA on PPY (**b**), DCF on PANI (**c**), and SA on PANI (**d**).

The adsorption of DCF and SA onto PPY and PANI polymeric chains was simulated by modeling the molecular interaction between the different molecules using Cerius^2^ software [28].The total energy of the system was calculated by considering all bonded, dispersive, and electrostatic energy terms. Amorphous cells comprising molecules of the contaminants and the adsorbents in different compositions were constructed at room temperature using Monte Carlo methods, resembling the experimental density of the systems by controlling the volume (V) of the 3D simulation boxes (Figure 2). Periodic boundary conditions ensured that the various simulated atoms experienced the forces they usually do in the bulk phase. The Ewald summation method was applied to account for all short- and long-range interactions. Following energy minimization and system equilibration, molecular dynamics techniques were used to generate trajectories 10 ns long at the NVT ensemble. Every simulation was repeated four times and used to perform the calculations of the enthalpy of mixing using the cohesive energy approach according to Equation (3):

ΔH_mix_/V = ϕ_1_ CED_1_ + ϕ_2_ CED_2_ − (ϕ_1_ + ϕ_2_) CED_12_ (3)

where ϕ_i_ refers to the mole fraction of component i and CED_i_ refers to the cohesive energy density of system i [29]. The calculated enthalpy of mixing was thus used to express the miscibility and interaction of the different systems since a negative enthalpy of mixing indicates a favorable interaction between the different simulated molecules and vice versa. 

## 3. Results and Discussion

### 3.1. Characterization of PPY-Coated and PANI-Coated Cotton Fabrics

**Figure 3:**
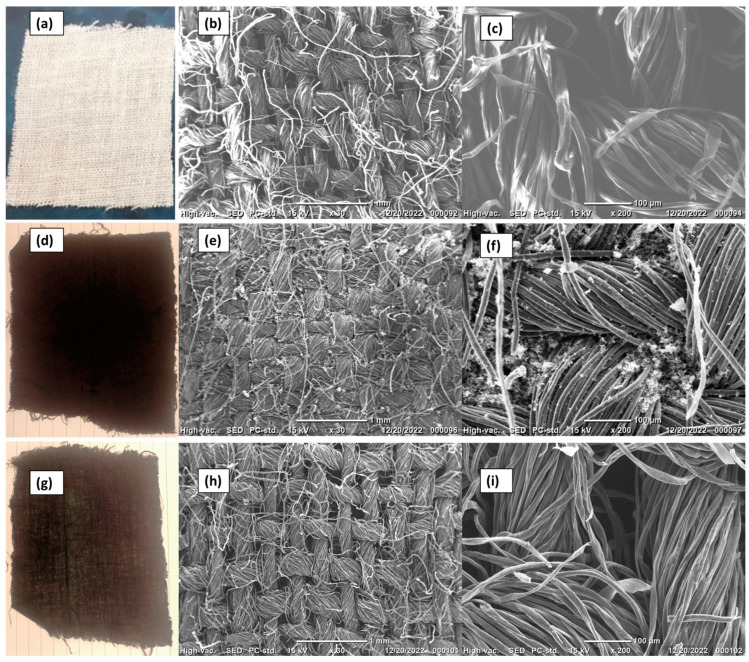
Photos and SEM images of cotton fabric (**a**–**c**), PPY-coated cotton fabric (**d**–**f**), and PANI-coated cotton fabric (**g**–**i**) at two different scales of 1 mm (**b**,**e**,**h**) and 100 µm (**c**,**f**,**i**).

The coating of the cotton fabrics with PPY or PANI can be seen by the naked eye, as shown in Figure 3a,d,g, which confirm the successful coating of cotton fabrics with PPY or PANI as the color of the original white Egyptian cotton fabrics was changed to a black-pigmented or dark-green-pigmented textile, respectively. Clearly, a homogeneous coating of PPY or PANI appears on the cotton fabric.

For a closer examination of the coating, SEM images were recorded for the bare cotton fabric (Figure 3b,c) and after PPY coating (Figure 3e,f) and PANI coating (Figure 3h,i) at two different scales. The images indicate the presence of PPY and PANI on the cotton threads in a uniform and homogenous coating. Due to the globular morphology of PPY, residual traces of the polymer can be seen on the cotton threads after the coating and washing processes. 

**Figure 4:**
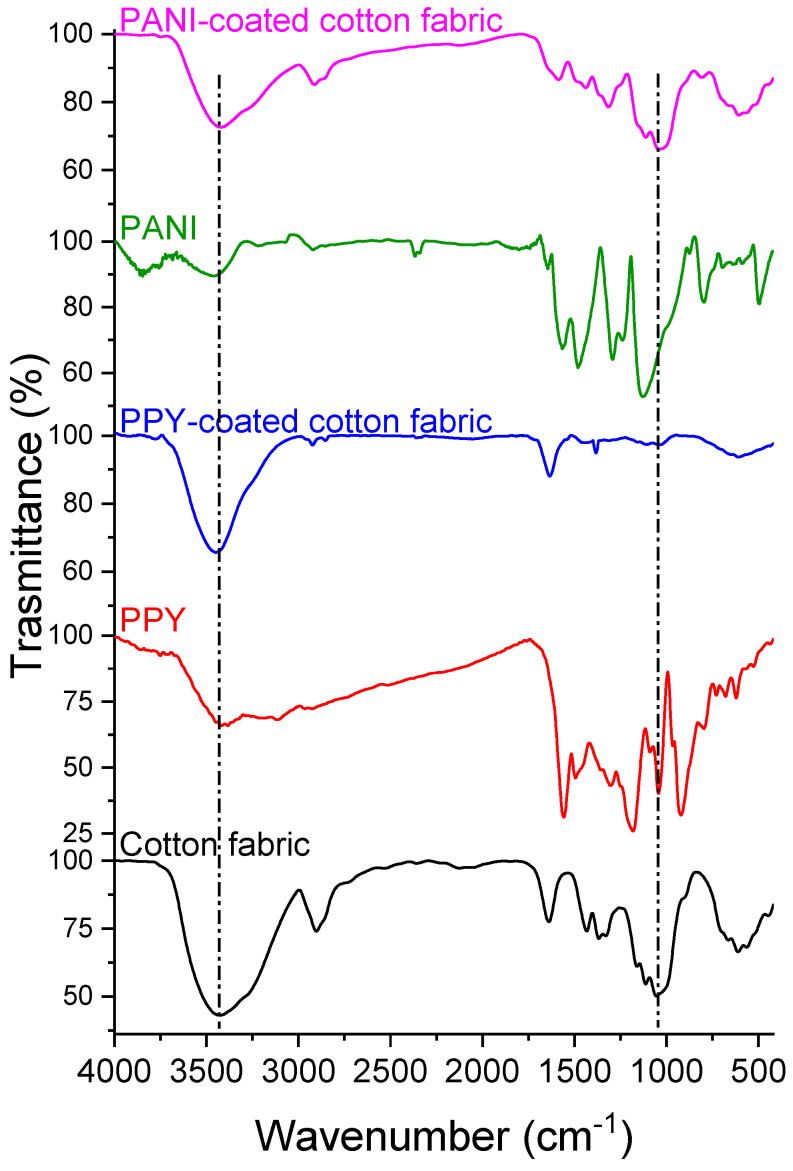
FTIR spectra of bare, PPY, PPY−coated cotton fabrics, PANI, and PANI−coated cotton fabrics.

The cotton fabric was further characterized by measuring its FTIR before and after it was coated with PPY, as shown in Figure 4. The cotton textile exhibits bands at 3425 cm^−1^ and 2897 cm^−1^ due to OH and CH symmetric stretching vibrations, respectively. The absorption at 1637 cm^−1^ can result from the absorbed moisture. The CH_2_ and CH symmetric bending vibrations appeared at 1433 cm^−1^ and 1367 cm^−1^, respectively. The band at 1055 cm^−1^ is assigned to CO stretching, while the band at 664 cm^−1^ is attributed to OH out-of-phase bending. After coating with PPY, some peaks became unclear, and other peaks were shifted. The band at 3449 cm^−1^ is attributed to NH in the pyrrole ring. The bands at 2918 cm^−1^ and 2858 cm^−1^ are due to CH_2_ and CH symmetric stretching in the five-membered pyrrole ring. The band at 1635 cm^−1^ is assigned to the ring stretching vibration of a C=C bond, while a C-H deformation vibration appeared at 1030 cm^−1^ [30,31]. The spectrum of the PANI-coated cotton textiles shows a band at 3421 cm^−1^ that is related to the stretching vibration of the secondary amine (N-H) in PANI. The peaks at 2917 cm^−1^ and 2858 cm^−1^ are attributed to CH_2_ and CH symmetric stretching in the aniline ring. The peaks at 1582 cm^−1^ and 1435 cm^−1^ are due to quinonoid and benzenoid ring stretching, respectively [32,33]. The peak at 1314 cm^−1^ is assigned to C-N stretching of the secondary aromatic amine, while the peaks at 1033 cm^−1^ and 612 cm^−1^ are ascribed to C-H bending vibrations in the aromatic ring.

### 3.2. Adsorption Studies

#### 3.2.1. Effect of pH

**Figure 5:**
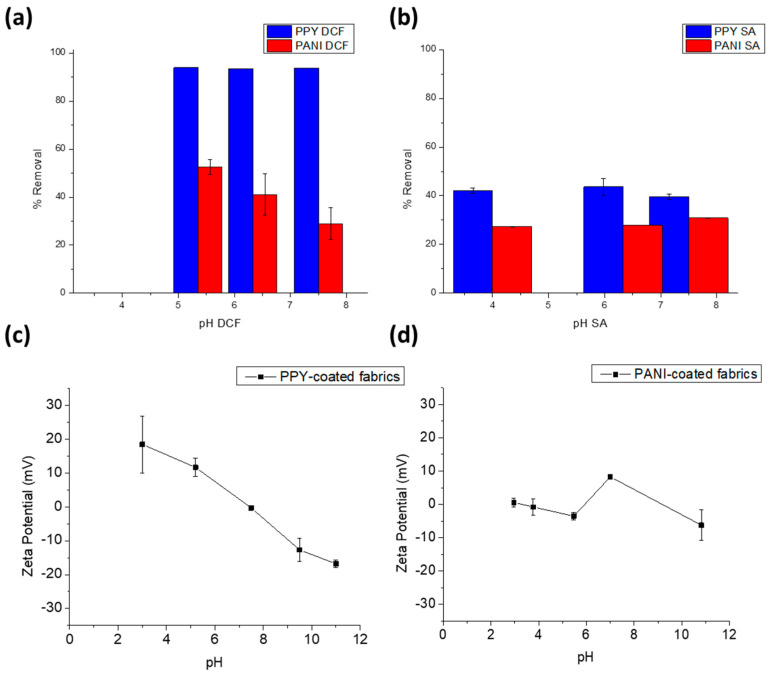
pH effect on DCF (**a**) and SA (**b**) adsorption onto 1 × 2 cm^2^ PPY−coated and PANI−coated fabrics. Adsorption was conducted for 50 ppm of DCF or SA at 25 ± 2 °C. Zeta potential curves for PPY−coated (**c**) and PANI−coated (**d**) fabrics. Error bars are expressed as mean values ± SD, *n* = 3.

Figure 5a,b show the effect of pH on the adsorption of DCF and SA by the PPY- and PANI-coated fabrics. In the investigated pH ranges of 5.3–7.5 for DCF and 4.0–7.5 for SA, no significant change (*p* > 0.05) can be observed in the percent removal of DCF or SA on either the PPY-coated or PANI-coated cotton fabrics. However, for PANI there is a slight increase in the percent removal of DCF from 28.9% to 41.0%, and to 52.5% with decreasing pH values from 7.5 to 6.33 and 5.35, respectively. A similar behavior was reported in the literature where a PPY/PANI composite film exhibited negligible change in the removal efficiency of chromium ions (Cr VI) with changing the pH from 4 to 10, which indicated that there was no competitive interaction between the hydrogen or hydroxyl ions and the chromium ions on the composite surface [34]. Therefore, pH 5.3 and pH 4 were selected in the current study as the respective working pHs for the adsorption of DCF and SA on the coated fabrics since they yield optimal removal efficiencies for both drugs, and meanwhile they are the normal pHs of the drug solutions without pH adjustment. The respective zeta potential measurements for the PPY- and PANI-coated fabrics depicted in Figure 5c,d reveal that both coated fabrics exhibited a slightly positive to almost neutral net surface charge at the working pHs [35]. On the other hand, the speciation diagrams of DCF and SA indicate that they are negatively charged at these pHs (www.chemicalize.com, accessed on 20 November 2022). Thus, it can be anticipated that the mechanisms of adsorption could involve physical electrostatic binding between the drugs and the oppositely charged fabrics. 

#### 3.2.2. Time Profiles and Kinetics

**Figure 6:**
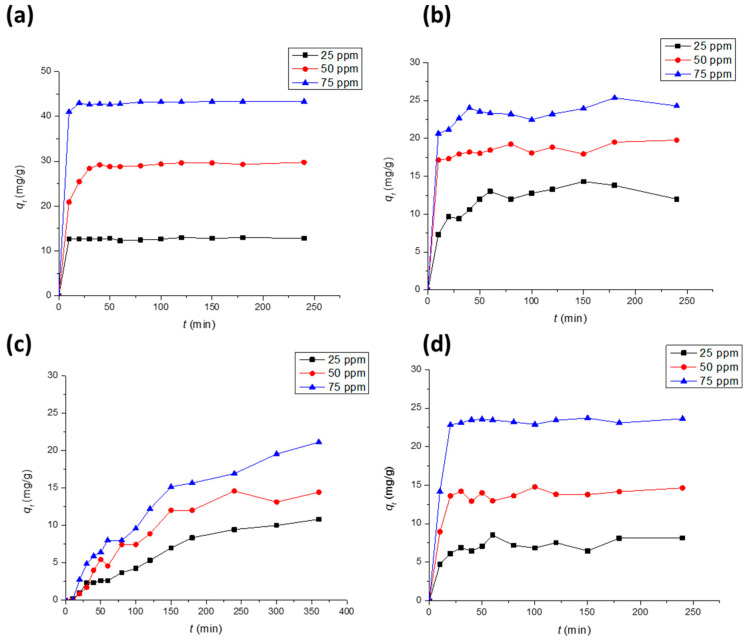
Uptake profiles of *q*_t_ vs. *t* for the adsorption of (**a**) DCF on (1 × 2 cm^2^) PPY-coated fabrics at pH 5.3, (**b**) DCF on PANI-coated fabrics at pH 5.3, (**c**) SA on PPY-coated fabrics at pH 4.0, and (**d**) SA on PANI-coated fabrics at pH 4.0, all at room temperature 25 ± 2 °C.

As shown in Figure 6, the effect of contact time on the adsorption of DCF and SA onto PPY-coated and PANI-coated fabrics at three different concentrations is investigated. The time profiles reach their equilibrium at about 20–30 min for all concentrations of DCF on the PPY-coated and PANI-coated fabrics and for all concentrations of SA on the PANI-coated fabric. However, for the SA adsorption on the PPY-coated fabric, equilibrium is barely attained at about 240, 240, and 300 min at the applied SA concentrations of 25, 50, and 75 ppm, respectively. Clearly, the equilibrium adsorption capacity increases with increasing the concentration owing to the increase in the driving force, which overcomes the mass transfer resistance [36].

To better understand the adsorption behavior and the contact time effect, kinetic studies were performed on the adsorption of 50 ppm of DCF on the 1 × 2 cm^2^ PPY-coated and PANI-coated fabrics at pH 5.3 and the adsorption of 50 ppm of SA on the 1 × 2 cm^2^ PPY-coated and PANI-coated fabrics at pH 4.0 and 25 °C. The most widely used kinetic models (Equations (4)–(6)) were applied [37]:

Pseudo-first-order model:

(4) $$\log(q_{e}-q_{t})=\log\left(q_{e}\right)-\frac{k_{1}t}{2.303}$$

Pseudo-second-order model:

(5) $$\frac{t}{\;q_{t}\;}=\frac{1}{k_{2}q_{e}^{2}}+\frac{1}{q_{e}}\;t$$

Intra-particle diffusion:

(6) $$q_{t}=k_{id}t^{0.5}+C$$

where *k*_1_, *k*_2_, and *k_id_* are the rate constants for the pseudo-first-order, pseudo-second-order, and intra-particle diffusion models, respectively, *q_e_* is the equilibrium adsorption capacity, and $q_{t}$ is the adsorption capacity at time *t,* whereas *C* is the film diffusion constant.

The above kinetic models were tested for their validity to predict the adsorption profiles for the systems investigated in the current work, and the kinetic parameters along with the correlation coefficient values (*R*^2^) are presented in Table 1 and Table S1. The adsorption of DCF on the PPY-coated and PANI-coated fabrics along with SA on the PANI-coated fabric followed the pseudo-second-order model, as evident from its higher *R^2^* values and lower *RMSE* (root mean square error) values as compared to the corresponding pseudo-first-order values, which indicates that adsorption took place via different mechanisms, i.e., surface reaction, film diffusion, and pore diffusion (Table 1, Figures S1 and S2). Previous work reported that adsorption onto PPY/multiwalled carbon nanotube composites and PANI powder also followed the pseudo-second-order model when tested for adsorption with DCF [38,39]. In the case of SA adsorption on the PPY-coated fabric, it followed the pseudo-first-order model, as indicated by its higher *R^2^* value of 0.9153 and lower *RMSE* compared to the relevant pseudo-second-order values (Table 1, Figures S1 and S2). Mathematically, it was stipulated by Ho and McKay (1999) that the pseudo-first-order model applies to the initial stages of adsorption [40]. This behavior is consistent with the kinetic profiles of SA-PPY adsorption, which barely approached equilibrium during the investigated time interval, and this implies that adsorption herein is still in the initial stage. Due to the impracticality of applying such a slow process, investigating longer time periods is not warranted. This behavior was also reported for SA on a resin with a distinctive double pore structure [41]. To further assess the mechanism of interaction, the kinetic profiles were modeled using the intra-particle diffusion model (Figure S3 and Table S1). The plot for DCF adsorption on the PPY-coated fabric (Figure S3a) demonstrates two stages. The first stage is presented by a line that passes through the point of origin (0,0), and this indicates that adsorption is controlled by pore diffusion, while the second stage is the equilibrium stage, where the adsorption capacity remains constant with time. The plot of DCF adsorption on PANI-coated fabrics (Figure S3b, Supplementary Information), on the other hand, exhibits two stages as well; however, the line representing the first stage has an intercept, which indicates that adsorption is governed by film diffusion. It is also possible that this stage is preceded by a pore-diffusion-controlled step, as in the case of DCF onto PPY-coated fabrics; however, it is an instantaneous step that could not be detected here, as it is anticipated to occur in less than 2 min. As for the adsorption of SA on PPY-coated fabrics, its plot (Figure S3c) manifests only one line passing through the origin, indicating pore-diffusion-controlled adsorption. Since the process is slow, the equilibrium stage is not shown within the investigated time interval. The adsorption plots of SA on PANI-coated fabrics (Figure S3d), however, show two stages corresponding to pore-diffusion-controlled adsorption followed by equilibrium. The intra-particle diffusion constant (*k*_id_) for SA adsorption on PANI-coated fabrics is higher than that on PPY-coated ones (Table S1), implying a faster rate of diffusion of SA into PANI-coated fabrics.

#### 3.2.3. Adsorption Isotherms

**Figure 7:**
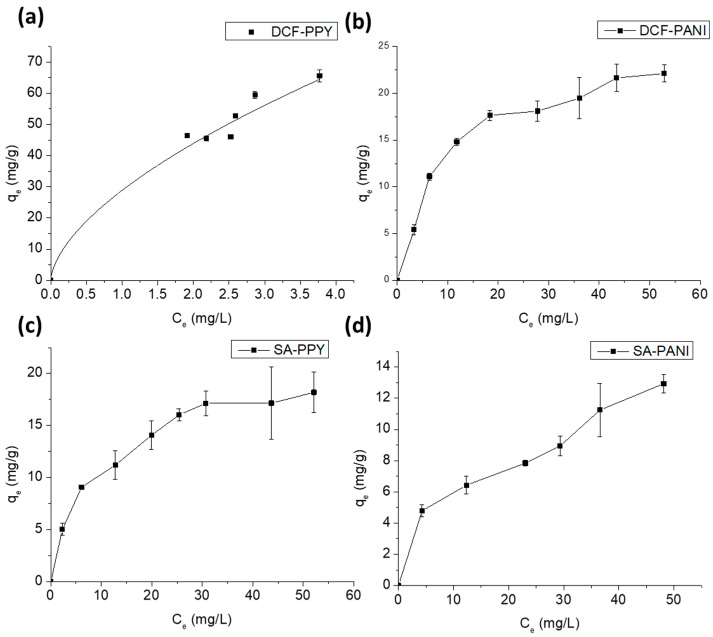
The *q_e_* vs. *C_e_* plots for (**a**) DCF-PPY at pH 5.3, (**b**) DCF-PANI at pH 5.3, (**c**) SA-PPY at pH 4.0, and (**d**) SA-PANI at pH 4.0. The adsorption was conducted for 4 h in the case of DCF adsorption and 5 h in the case of SA adsorption using 1 × 2 cm^2^ fabrics at room temperature 25 °C. Error bars are expressed as mean values ± SD, *n* = 3.

Figure 7 shows the *q_e_* (equilibrium adsorption capacity) vs. *C_e_* (equilibrium concentration) plots for the adsorption of DCF and SA on the PPY-coated and PANI-coated fabrics. Clearly, the shapes of the adsorption isotherms are hyperbolic for DCF on the PANI-coated fabric as well as SA on the PPY-coated fabric. To predict the adsorption behavior, the most applied adsorption isotherm models (Equations (7) and (8)) were examined [42].

Langmuir isotherm:

(7) $$\frac{C_{e}}{q_{e}}=\frac{C_{e}}{q_{m}}+\frac{K_{d}}{q_{m}}$$

where *q_m_* is the maximum adsorption capacity and *K_d_* is the Langmuir desorption constant.

Freundlich isotherm:

(8) $$q_{e}=K_{f}C_{e}^{\frac{1}{n}}$$

where *K_f_* is a Freundlich constant related to the adsorption capacity and 1/*n* is related to the degree of heterogeneity.

By fitting the adsorption isotherms to Langmuir and Freundlich models, it was deduced that DCF adsorption on the PPY-coated fabric and SA adsorption on the PANI-coated fabric obeyed the Freundlich isotherm, which gave higher *R^2^* values and comparable or lower *RMSE* values relative to their corresponding Langmuir values, thus confirming multi-layer adsorption (Table 2, Figures S4 and S5), while the adsorption of DCF on the PANI-coated fabric and the adsorption of SA on the PPY-coated fabric obeyed the Langmuir isotherm, indicating a monolayer adsorption. The 1/*n* constant for Freundlich was less than 1, indicating a favorable adsorption [43]. 

#### 3.2.4. Parameters Affecting the Adsorption Process

**Figure 8:**
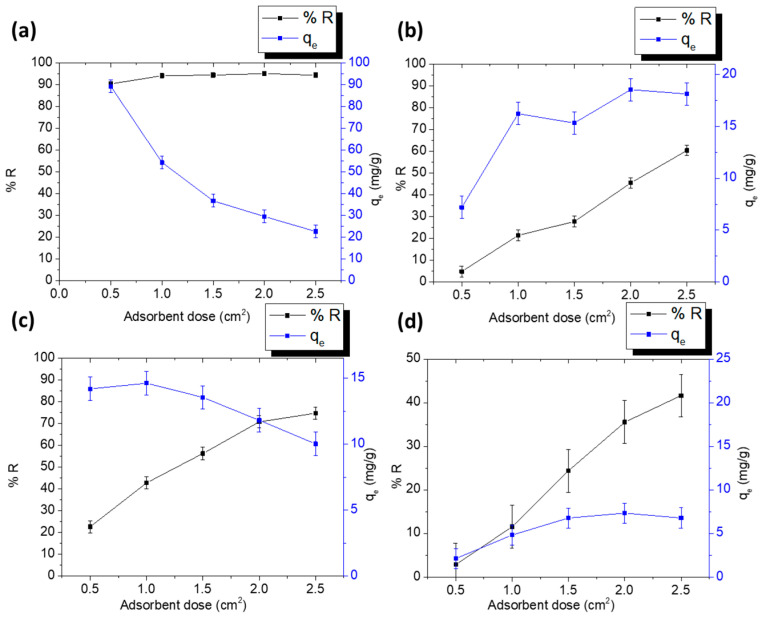
Adsorbent dose effect on %removal and *q_e_* for (**a**) DCF-PPY at pH 5.3, (**b**) DCF-PANI at pH 5.3, (**c**) SA-PPY at pH 4.0, and (**d**) SA-PANI at pH 4.0. Adsorption was conducted for 4 h in case of DCF adsorption on PPY-coated and PANI-coated fabrics and SA on PANI-coated fabrics, and 5 h for SA on PPY-coated fabrics using 50 ppm DCF and 25 ppm SA at room temperature 25 °C. Mean values ± SD, *n* = 3.

Figure 8 shows the effect of increasing the area of the coated fabrics on the percent removal and *q_e_* of DCF and SA onto the PPY-coated and PANI-coated fabrics. It is evident from Figure 8a for the adsorption of DCF on the PPY-coated fabric that *q_e_* decreased from 89.2 mg/g to 22.6 mg/g with increasing the dose from 0.5 to 2.5 cm^2^, indicating that the drug molecules were not sufficient to occupy all the adsorbent sites [44], whereas the percent removal encountered a slight increase from about 90 to 94% as the adsorption sites increased [45]. Similarly, the adsorption of SA on the PPY-coated fabric (Figure 8c) exhibited an increase in the percent removal from 22.6% to 74.7% and a decrease in *q_e_* from 14.18 mg/g to 10.01 mg/g upon increasing the dose from 0.5 to 2.5 cm^2^ [46]. However, in the case of the adsorption of DCF and SA on the PANI-coated fabrics (Figure 8b,d), both the percent removal and *q_e_* increased with increasing the dose owing to the increase in the adsorption sites. Similar behavior was observed with functionalized PVA films that were used for the adsorption of Congo Red from aqueous solutions [47].

**Figure 9:**
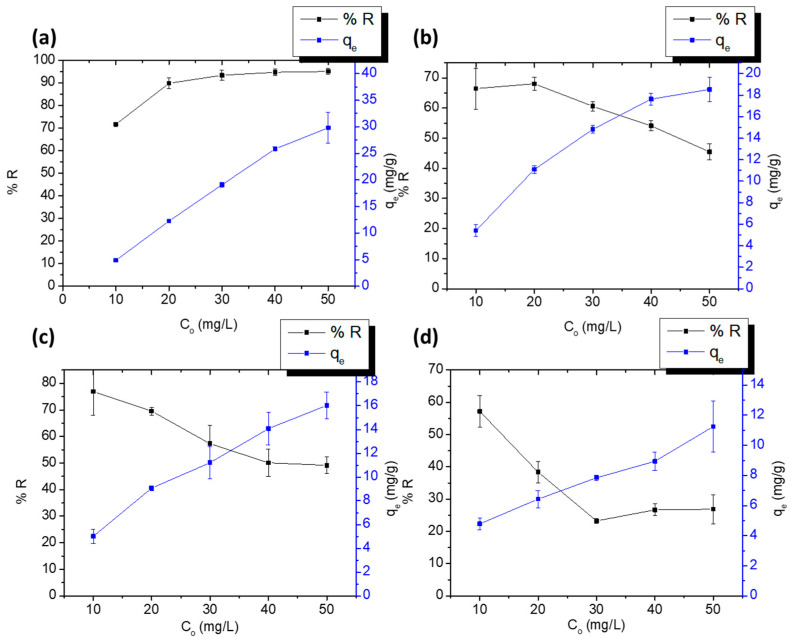
The effect of initial concentration on the adsorption of DCF on PPY-coated cloth (**a**), DCF on PANI-coated cloth (**b**), SA on PPY-coated cloth (**c**), and SA on PANI-coated cloth (**d**) in the range of 10–50 ppm over 1 × 2 cm^2^ PPY-coated and PANI-coated fabrics at pH 5.3 for DCF and 4.0 for SA, and at room temperature 25 °C. Error bars are expressed as mean values ± SD, *n* = 3.

Figure 9 shows the effect of the initial concentration of DCF and SA on the percent removal and *q_e_* of the PPY-coated and PANI-coated fabrics. As can be observed, *q_e_* increased with increasing the initial concentration in all cases, which could be ascribed to the increase in the concentration gradient and the decrease in mass transfer resistance [48]. Regarding the percent removal, it decreased with increasing the concentration in all cases except for the adsorption of DCF on the PPY-coated fabric. The former decline in removal could be owed to the occupation of most of the sites, as the adsorbent dose is constant [49]. For the adsorption of DCF on the PPY-coated fabric, however, the removal increased slightly with concentration then flattened out, probably due to the saturation of active sites. These results are similar to those reported in the literature for the adsorption of anionic dyes on a PANI/γ-alumina nanocomposite [50]. 

**Figure 10:**
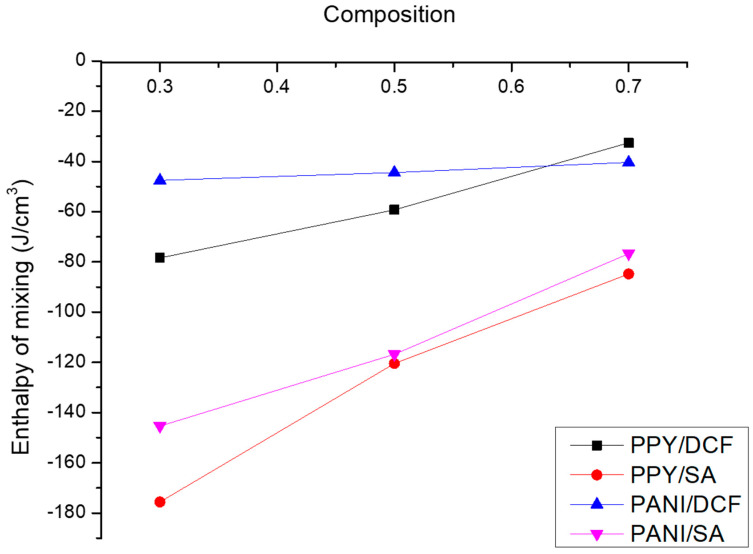
Effect of the sorbent/sorbate ratio on the simulated enthalpy of mixing.

The above observations could also be confirmed by modeling the enthalpy of mixing for the different sorbent/sorbate systems using Equation (3) [28,29] and as shown in Figure 10. Table 3 shows the interaction between the polymer sorbents and the contaminants given in terms of the total cohesive energy densities and its breakdown into van der Waals (non-coulombic) and electrostatic (coulombic) contributions to the cohesive energy density.

The values of the cohesive energy densities shown in Table 3 indicate that the interaction between the polymer chains and the contaminant molecules is driven by both non-coulombic interactions arising from van der Waals and π-π interactions and coulombic interactions between the charged groups on the different molecules. It is also obvious from the table that electrostatic forces are more sensitive to changes in the polymer composition than van der Waals forces. The results also show that the cohesive forces are higher for DCF and SA adsorption on the PPY-coated fabric than on the PANI-coated ones, which is consistent with the experimental findings that demonstrated higher removal efficiencies on the PPY-coated fabrics.

It is obvious from the above figure that for all of the polymer/contaminant systems under investigation, the negative enthalpy of mixing combined with a positive entropy of mixing, resulting from the increased randomness upon mixing, would give rise to a negative free energy of mixing and thus a favorable miscibility between the different substrates, therefore adding to the final adsorption and removal of the contaminants. It is also clear that the higher negativity values for the enthalpy of mixing SA with both PANI and PPY molecules would indicate a more favorable miscibility for these systems over those of DCF. However, the experimental results indicated less adsorption of SA, which leads to the conclusion that the adsorption is not only dependent on the interaction between the different molecules but rather on other factors such as steric hinderance, molecular geometry and spatial orientation, and polymer morphology.

## 4. Regeneration

**Figure 11:**
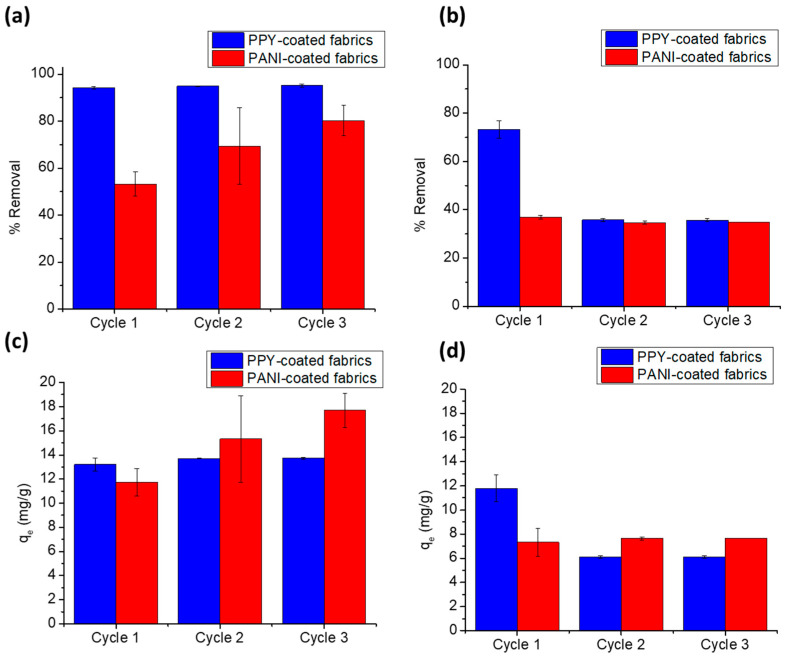
Regeneration study of PPY- and PANI-coated fabrics using 0.1 N HCl for DCF and 0.1 N NaOH for SA as desorbing agents after adsorption of (**a**,**c**) 25 ppm DCF at pH 5.3 on 1 × 2 cm^2^ PPY- and PANI-coated fabrics or (**b**,**d**) 25 ppm SA at pH 4.0 on 1 × 2 cm^2^ PPY- and PANI-coated fabrics. Panels (**a**,**b**) show the regeneration study in terms of %removal, while panels (**c**,**d**) present the regeneration study in terms of adsorption capacity. Error bars represent mean values ± SD, *n* = 3.

Figure 11 shows the results of the regeneration study of PPY-coated and PANI-coated fabrics using 0.1 N HCl or 0.1 N NaOH after the adsorption of 25 ppm DCF and SA. It is clearly shown that the percent removal and *q_e_* kept steady through the three cycles in the case of the adsorption of DCF on the PPY-coated cloth (Figure 11a,c) and SA on the PANI-coated cloth (Figure 11b,d). On the other hand, the percent removal and *q_e_* decreased abruptly (Figure 11b,d) by more than 50% in the case of SA adsorption on the PPY-coated cloth. This could be attributed to the stronger binding of SA to PPY- than to PANI-coated fabrics, as manifested in its higher CED values and the low *K*_d_ of its monolayer adsorption on the PPY-coated fabric in contrast to its multi-layer adsorption on the PANI-coated fabric. However, the percent removal and *q_e_* increased in the case of DCF adsorption on the PANI-coated fabric, which might be due to the deformation of pores leading to their increased size and ability to adsorb more drug molecules by virtue of acid erosion [51]. 

## 5. Adsorption Mechanism

The zeta potential measurements showed that the fabrics are positively charged to neutral while the drugs are negatively charged as per their speciation diagrams. This implies that possible electrostatic interactions can take place between DCF or SA and the fabrics, along with other physical interactions such as π-π stacking and van der Waals forces. The π-π stacking is likely to occur between the DCF or SA rings on the one hand and the benzenoid or quinonoid rings of PPY or PANI on the other hand. This was further confirmed by the modeling calculations, where the cohesive energy density values indicated that both electrostatic attractions and van der Waals interactions took place. Similar mechanisms were found with DCF adsorption on PPY-doped GO/COF-300 nanocomposites [52] and PANI nanofibers [53].

## 6. Comparison with Literature

Table 4 shows a list of some of the reported adsorbents and their maximum adsorption capacities (*q_m_*) [54,55,56]. Both PPY- and PANI-coated fabrics show superior adsorption capacities toward SA as compared to chitosan/xylan-coated magnetite and a comparable adsorption capacity for DCF relative to poly(acrylonitrile-*co*-ethylene glycol dimethacrylate-*co*-vinylbenzyl chloride). However, their capacities are less than that reported for phenyl-phosphate-based porous organic polymers with DCF and phosphorus-based hyper-cross-linked polymers with SA, which is understandable in view of the highly porous structure of these adsorbents as they are based on phosphate and quaternary phosphonium groups which have a high tendency to adsorb anions; nevertheless, they require sophisticated organic synthesis procedures.

## 7. Conclusions

Cotton fabrics were successfully coated with PPY and PANI, as confirmed by the SEM and FTIR measurements. The fabrics, when applied in the adsorption of DCF and SA, showed excellent percent removal, especially for DCF on the PPY-coated fabric, which reached removal efficiencies above 90%. Adsorption was suggested to occur via electrostatic attractions and van der Waals and π-π stacking, as inferred from the molecular modeling simulations. Adsorption followed the Langmuir isotherm in the cases of DCF on the PANI-coated fabric and SA on the PPY-coated fabric, where *q_m_* was about 26 and 21 mg/g, respectively. It followed Freundlich with DCF on the PPY-coated fabric and SA on the PANI-coated fabric. Kinetics followed the pseudo-second-order model in all cases except for SA adsorption on the PPY-coated fabric, which showed the slowest kinetics among the tested sorbate/adsorbent combinations. However, this combination demonstrated the highest binding strength, as indicated by its highest CED values. The high binding strength and the Langmuirian-type adsorption imply a predominance of chemisorption, which is a slow process. The adsorption of DCF on PPY-coated fabrics was, however, faster than SA, although they are both controlled by pore diffusion. This is probably since it is a multi-layer adsorption and because DCF possesses two aromatic rings that act as binding sites for possible π-π stacking. Moreover, DCF adsorbed on the PANI-coated fabric faster than on the PPY-coated fabric, and its adsorption was faster than that of SA on the PANI-coated fabric, probably since its adsorption is governed by external film diffusion. The fabrics were regenerated for three cycles using HCl and NaOH. Thus, the developed fabrics provide a facile, rapid, and efficient adsorption process due to their ease of handling and separation during operation as well as their functionality in removing different contaminants. To the best of our knowledge, there were no previous reports on applying the prepared adsorbents on the adsorption of pharmaceutical drugs in the literature. Also, the prepared adsorbents showed competitive behavior over those reported in the literature [21]. Future work might extend to applying adsorption experiments to the tested contaminants or other contaminants, either in simulated or real wastewater, to study the adsorption behavior in multi-component systems.

## Figures and Tables

**Table 1:** Parameters for the kinetic modeling studies for 50 ppm DCF and SA at pH 5.3 and 4.0, respectively. using 1 × 2 cm^2^ cut fabrics.

	Pseudo-First-Order Model				Pseudo-Second-Order Model			
	*q_e_,* mg g^−1^	*k*_1_, min^−1^	*R* ^2^	*RMSE*	*q_e_,* mg g^−1^	*k*_2_, g mg^−1^ min^−1^	*R* ^2^	*RMSE*
DCF-PPY	4.86	0.027	0.8336	0.229	30.03	0.01270	0.9998	0.035
SA-PPY	15.02	0.0207	0.9321	0.192	23.92	0.00021	0.8475	1.449
DCF-PANI	2.62	0.0094	0.9011	0.045	19.08	0.02738	0.9998	0.021
SA-PANI	3.76	0.0117	0.8321	1.822	14.64	0.01577	0.9977	0.194

**Table 2:** Parameters for Langmuir and Freundlich adsorption isotherms.

	Langmuir				Freundlich			
	*q_m_,* mg/g	*K_d_,* L/mg	*R* ^2^	*RMSE*	1/*n*	*K_f_*, (mg/g) × (L/mg)^n^	*R* ^2^	*RMSE*
DCF-PPY	136.99	5.00	0.8314	3.20	0.5836	30.52	0.9212	3.17
DCF-PANI	26.25	10.51	0.9899	0.86	0.4544	4.05	0.8942	1.78
SA-PPY	21.01	8.649	0.9930	0.66	0.4114	3.92	0.9666	1.06
SA-PANI	16.07	17.27	0.8815	1.09	0.3966	2.49	0.9404	0.76

**Table 3:** Cohesive energy densities of the sorbent/sorbate systems with different compositions.

CED (J cm^−3^)									
System	Total			Van Der Waals			Electrostatic		
Ratio	30/70	50/50	70/30	30/70	50/50	70/30	30/70	50/50	70/30
PPY/DCF	344.8	345.4	338.5	270.2	283.4	290.5	60.26	53.35	40.38
PANI/DCF	313.9	330.6	346.3	218	272.7	296.9	53.91	50.51	41.29
PPY/SA	518.4	461.2	423.5	291.2	299.9	309	217.9	154.7	106.8
PANI/SA	488.2	457.5	415.4	283.2	294.2	304.8	204.1	155.8	102.8

**Table 4:** Adsorption capacities of some of the reported adsorbents.

Adsorbent	Contaminant	*q_m_,* (mg/g)	Reference
Phenyl-phosphate-based porous organic polymers	DCF	217 (Langmuir)	[54]
Chitosan/xylan-coated magnetite nanoparticles	SA	11.34 (Freundlich)	[10]
Poly(acrylonitrile-*co*-ethylene glycol dimethacrylate-*co*-vinylbenzyl chloride)	DCF	61 (Langmuir)	[55]
Phosphorus based hyper cross-linked polymers	SA	369 (Langmuir)	[56]
PPY-coated fabrics	DCF	65.61 (Freundlich)	This work
	SA	21.01 (Langmuir)	
PANI-coated fabrics	DCF	26.25 (Langmuir)	This work
	SA	12.92 (Freundlich)	

## Data Availability

Available within manuscript and Supplementary File.

## Supplementary Materials

The following supporting information can be downloaded at: https://www.mdpi.com/article/10.3390/polym15173563/s1, Figure S1: Pseudo-first order kinetic plots for the adsorption of (a) DCF on (1 × 2 cm^2^) PPY at pH 5.3, (b) DCF on PANI at pH 5.3 (c) SA on PPY at pH 4.0, and (d) SA on PANI at pH 4.0, all at room temperature 25 ± 2 °C and initial concentration of 50 ppm; Figure S2: Pseudo-second order kinetic plots for the adsorption of (a) DCF on (1 × 2 cm^2^) PPY at pH 5.3, (b) DCF on PANI at pH 5.3 (c) SA on PPY at pH 4.0, and (d) SA on PANI at pH 4.0, all at room temperature 25 ± 2 °C and initial concentration of 50 ppm; Figure S3: Intra-particle diffusion plots for the adsorption of (a) DCF on (1 × 2 cm^2^) PPY at pH 5.3, (b) DCF on PANI at pH 5.3 (c) SA on PPY at pH 4.0, and (d) SA on PANI at pH 4.0, all at room temperature 25 ± 2 °C and initial concentration of 50 ppm; Figure S4: Plots of Langmuir model for the adsorption of (a) DCF on (1 × 2 cm^2^) PPY at pH 5.3, (b) DCF on PANI at pH 5.3 (c) SA on PPY at pH 4.0, and (d) SA on PANI at pH 4.0, all at room temperature 25 ± 2 °C; Figure S5: Plots of Freundlich model for the adsorption of (a) DCF on (1 × 2 cm^2^) PPY at pH 5.3, (b) DCF on PANI at pH 5.3 (c) SA on PPY at pH 4.0, and (d) SA on PANI at pH 4.0, all at room temperature 25 ± 2 °C; Table S1: Intra-particle diffusion parameters at 50 ppm of contaminant.
