# Supplementary material for: Polypyrrole- and Polyaniline-Coated Cotton Fabrics as Efficient Adsorbents for the Pharmaceutical Water Contaminants Diclofenac and Salicylic Acid
Source: Polymers (Basel). 2023 Aug 28;15(17):3563. doi: 10.3390/polym15173563 (PMC10490524; doi:10.3390/polym15173563)
Supplement: Supplementary file 1 [file polymers-15-03563-s001.zip › polymers-2568520-SI.pdf]

Supplementary Information

Polypyrrole- and Polyaniline-Coated Cotton Fabrics as Efficient Adsorbents for the Pharmaceutical Water Contaminants Diclofenac and Salicylic Acid

Hebatullah H. Farghal [1], Samar H. Tawakey [2], Wael A. Amer [2,3], Mohamad M. Ayad [2,4], Tarek M. Madkour [1] and Mayyada M. H. El-Sayed [1,*]

[1] Department of Chemistry, School of Sciences and Engineering, The American University in Cairo, AUC Avenue, P.O. Box 74, New Cairo, Cairo 11835, Egypt; hebatullahfarghal@aucegypt.edu (H.H.F.); tarekmadkour@aucegypt.edu (T.M.M.)

[2] Chemistry Department, Faculty of Science, Tanta University, Tanta 31527, Egypt; samar.h.tawakey@gmail.com (S.H.T.); wael.amer@science.tanta.edu.eg (W.A.A.); mohamed.ayad@science.tanta.edu.eg or mohamad.ayad@ejust.edu.eg (M.M.A.)

[3] Department of Chemistry, College of Science, University of Bahrain, Sakhir 32038, Bahrain

[4] Institute of Basic and Applied Sciences, Egypt-Japan University of Science and Technology, New Borg El-Arab City, Alexandria 21934, Egypt

* Correspondence: mayyada@aucegypt.edu; Tel.: +20-2615-2564; Fax: +20-2795-7565

Kinetics

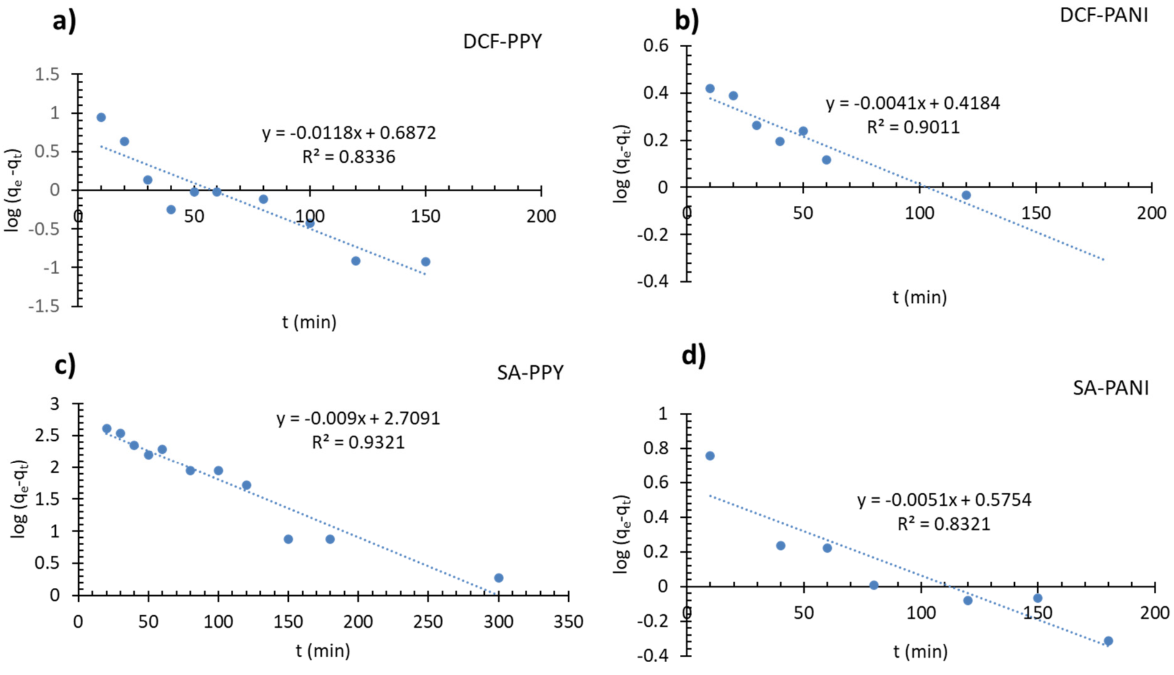

Figure S1. Pseudo-first order kinetic plots for the adsorption of (a) DCF on (1×2 cm^2) PPY at pH 5.3, (b) DCF on PANI at pH 5.3 (c) SA on PPY at pH 4.0, and (d) SA on PANI at pH 4.0, all at room temperature 25±2 °C and initial concentration of 50 ppm.

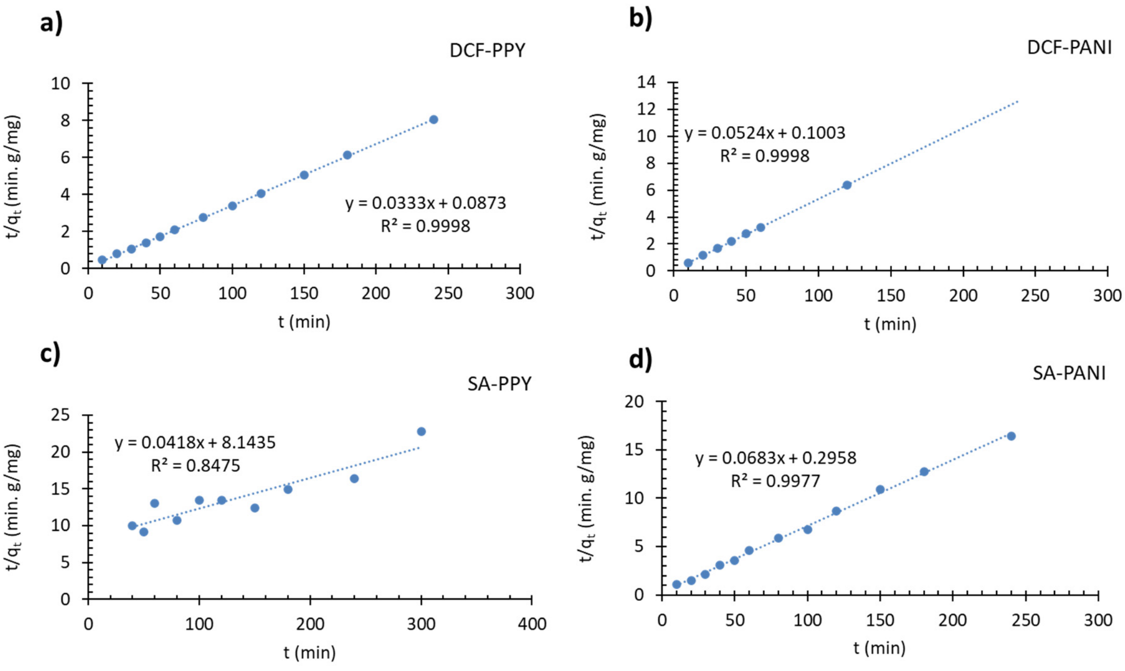

Figure S2. Pseudo-second order kinetic plots for the adsorption of (a) DCF on (1×2 cm^2) PPY at pH 5.3, (b) DCF on PANI at pH 5.3 (c) SA on PPY at pH 4.0, and (d) SA on PANI at pH 4.0, all at room temperature 25±2 °C and initial concentration of 50 ppm.

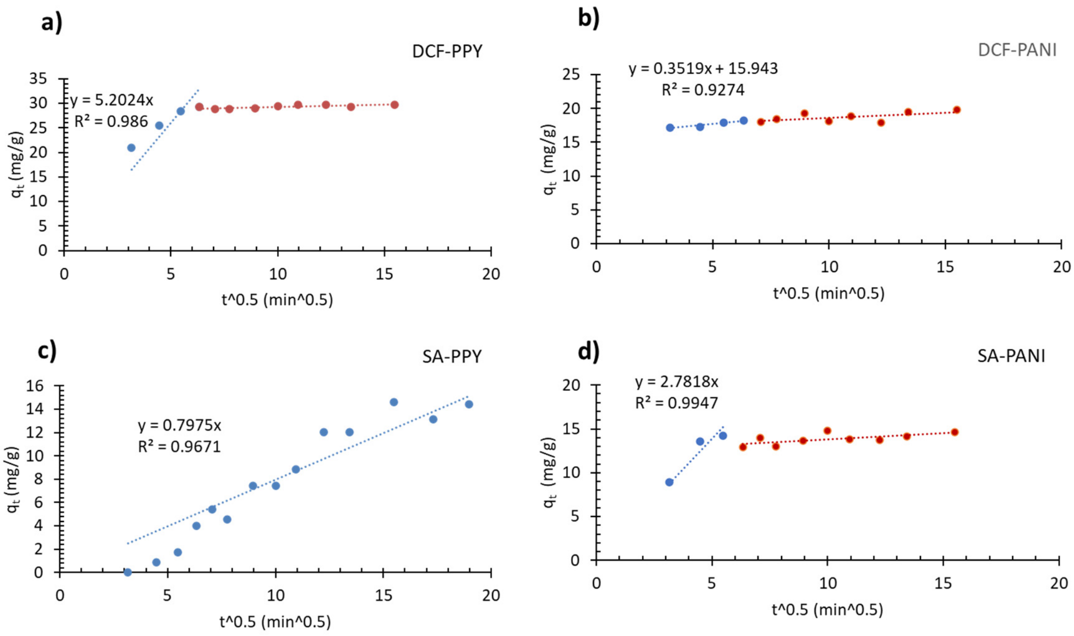

Figure S3. Intra-particle diffusion plots for the adsorption of (a) DCF on (1×2 cm^2) PPY at pH 5.3, (b) DCF on PANI at pH 5.3 (c) SA on PPY at pH 4.0, and (d) SA on PANI at pH 4.0, all at room temperature 25±2 °C and initial concentration of 50 ppm.

Table S1. Intra-particle diffusion parameters at 50 ppm of contaminant

	C, $mg.g^{-1}$	k_{id}, $mg.g^{-1}.min^{-0.5}$	R^2
**DCF-PPY**	0	5.202	0.9860
**DCF-PANI**	15.943	0.3519	0.9274
**SA-PPY**	0	0.797	0.9671
**SA-PANI**	0	2.7818	0.9947

Equilibrium isotherms

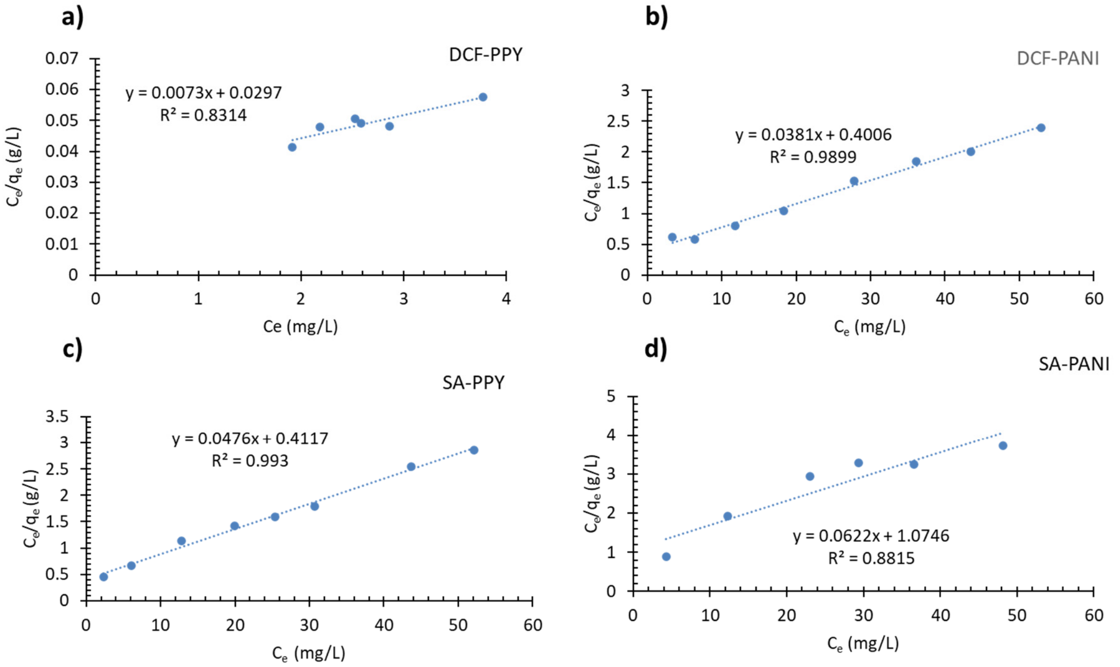

Figure S4. Plots of Langmuir model for the adsorption of (a) DCF on (1×2 cm^2) PPY at pH 5.3, (b) DCF on PANI at pH 5.3 (c) SA on PPY at pH 4.0, and (d) SA on PANI at pH 4.0, all at room temperature 25±2 °C.

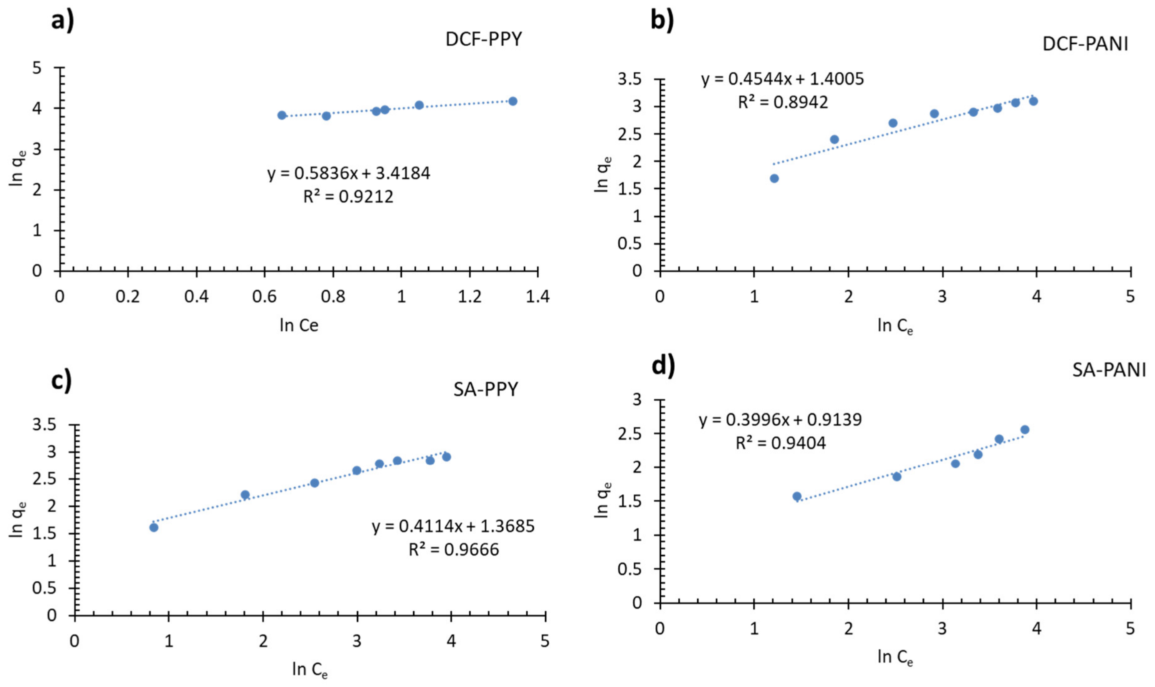

Figure S5. Plots of Freundlich model for the adsorption of (a) DCF on (1×2 cm^2) PPY at pH 5.3, (b) DCF on PANI at pH 5.3 (c) SA on PPY at pH 4.0, and (d) SA on PANI at pH 4.0, all at room temperature 25±2 °C.
